# Supplementary material for: Prussian Blue Nanozymes with Enhanced Catalytic Activity: Size Tuning and Application in ELISA-like Immunoassay
Source: Nanomaterials (Basel). 2022 May 10;12(10):1630. doi: 10.3390/nano12101630 (PMC9147909; doi:10.3390/nano12101630)
Supplement: Supplementary file 1 [file nanomaterials-12-01630-s001.zip › nanomaterials-1676008-supplementary.pdf]

Supplementary Materials

Prussian Blue Nanozymes with Enhanced Catalytic Activity: Size Tuning and Application in ELISA-Like Immunoassay

Pavel Khramtsov [1,2,*], Maria Kropaneva [1,2], Artem Minin [3,4], Maria Bochkova [1,2], Valeria Timganova [2], Andrey Maximov [5], Alexey Puzik [6,7,8,9], Svetlana Zamorina [1,2] and Mikhail Rayev [1,2]

1 Faculty of Biology, Perm State University, 614068 Perm, Russia; kropanevamasha@gmail.com (M.K.); krasnykh-m@mail.ru (M.B.); mantissa7@mail.ru (S.Z.); mraev@iegm.ru (M.R.)
2 Lab of Ecological Immunology, Institute of Ecology and Genetics of Microorganisms, 614081 Perm, Russia; timganovavp@gmail.com
3 Lab of Applied Magnetism, M.N. Mikheev Institute of Metal Physics of the UB RAS, 620108 Yekaterinburg, Russia; calamatica@gmail.com
4 Faculty of Biology and Fundamental Medicine, Ural Federal University Named after The First President of Russia B.N. Yeltsin, 620002 Yekaterinburg, Russia
5 Department of Analytical Chemistry and Expertise, Faculty of Chemistry, Perm State University, 614068 Perm, Russia; htb03starosta@gmail.com
6 Faculty of Geology, Department of Mineralogy and Petrography, Perm State University, 614068 Perm, Russia; alex.puzik@mail.ru
7 Core Facilities and Lab of Hydrochemical Analysis, Perm State University, 614068 Perm, Russia
8 Lab of Technological Mineralogy, Institute of Natural Science, Perm State University, 614068 Perm, Russia
9 Lab of Biogeochemistry of Technogenic Landscapes, Perm State University, 614068 Perm, Russia
* Correspondence: khramtsovpavel@yandex.ru; Tel.: +7-342-280-77-94

Reagents and instrumentation

Iron (III) chloride hexahydrate and 3,3',5,5'-tetramethylbenzidine dihydrochloride (TMB) were from AppliChem (USA); potassium hexacyanoferrate (II) trihydrate, potassium hexacyanoferrate (III), casein, gelatin A 180 bloom, Proclin-950 were from Sigma-Aldrich (USA). Tween-20, glutaraldehyde, citric acid, oxalic acid, glycine, sodium phosphate, sodium bicarbonate, and glycerol were from ITW (USA). Potassium hydroxide, sodium hydroxide, sulphuric acid, and hydrochloric acid were from Reakhim (Russia). Recombinant protein G from *Streptococcus sp.* was kindly provided by Dr. Tatyana Gupalova, Institute of Experimental Medicine (St.-Petersburg, Russia). Tetanus toxoid was from Mikrogen (Russia). WHO standard of human anti-tetanus IgG (TE-3) was from NIBSC (UK). PSA and anti-PSA monoclonal antibodies (clones 3A6 and 1A6) were obtained from Bialexa (Russia). 96-well polystyrene plates (high binding) were from SPL Life Sciences (South Korea) Dialysis tubing (cellulose membrane; 10,000 MWCO) was from Thermo Scientific (USA). Horseradish peroxidase (RZ 3.0; 307 U/mg) was from VWR (USA).

Buffers for immunoassay. 0.2 M carbonate buffer, pH 9.6; phosphate buffer (PB, 10 mM Na_2HPO_4, 10 mM NaH_2PO_4, and 0.05% Proclin-950, pH 7) and PBT (PB+0.1% Tween-20). Substrate buffer: 5 mM citrate-phosphate buffer, pH 4 and 0.1 M citrate-phosphate buffer, pH 5.

Instrumentation. Stat Fax 2600 microplate washer was from Awareness Technology (USA). Multiskan Sky UV-Vis Reader and iCAP 6500 Duo ICP-MS were from Thermo Scientific (USA). ZetaSizer NanoZS particle analyzer was from Malvern (UK). Aurora M90 ICP-MS was from Bruker Corp. (USA). Peristaltic pump P-1 was from Pharmacia (Sweden). VCX-130 ultrasonic processor was from Sonics & Materials (USA).

Methods: characterization of nanoparticles

Size, zeta-potential, and absorbance measurement

The zeta-potential and diameter of the nanoparticles were measured by M3-PALS (mixed mode measurement-phase analysis light scattering) technique and DLS respectively. For size measurements, ‘artificial peroxidase’ nanoparticles were diluted 1:375 (2 μl of nanoparticle suspension + 750 μl of H_2O) in deionized water; prussian blue

nanoparticles prepared by traditional method and 'artificial peroxidase' nanoparticles synthesized in the presence of 1 M HCl, KCl were diluted 1:125 (6 µl of nanoparticle suspension + 750 µl of H_2O) in deionized water. Plastic cuvettes (10 × 4 × 45 mm) were used for measurements. Measurements were performed at the scattering angle of 173° in auto mode. A general-purpose model was used to fit the data. Z-average diameters are reported throughout the article unless otherwise stated. The concentration of nanoparticles was preliminary optimized (Figure S17).

For zeta-potential measurement, samples were diluted 1:100 in deionized water with the addition of 1 mM of NaCl and 0.05% Tween-20 (7 µl of nanoparticle suspension + 700 µl of water). The pH of the solution was adjusted to 6 using 1 M NaOH. Plastic cuvettes (10 × 10 × 45 mm) were used for measurements. Measurements were performed in auto mode using a Dip Cell electrode (Malvern, UK). All measurements were done in triplicate.

To assess the A_{700} of the resulting suspensions, prussian blue nanoparticles were diluted 1:21 in deionized water (5 µl of particles + 100 µl H_2O) in 96-well plates; then, spectra at 400-1000 nm were measured.

Elemental analysis

The concentration of iron and potassium in the obtained samples was estimated by inductively coupled plasma atomic emission spectroscopy (ICP-MS).

Gravimetric analysis

Porcelain crucibles were heated at +140 °C to constant weight. Then samples were poured into them in a volume of 1 mL or 3 mL (**T**, **T25C**, and **T/dw/rt**). The water was evaporated at +95 °C, after which the samples were dried to constant weight at +140 °C.

Evaluation of the peroxidase-like catalytic activity of nanozymes

The specific activity of prussian blue nanoparticles and horseradish peroxidase was measured according to the protocol proposed by Jiang et al. [**1**], with modifications. Measurements were performed in 4 ml plastic cuvettes (10 mm path length). All solutions were kept at +37 °C in the water bath before mixing. Nanoparticles (or enzyme) were added to 1 mM Na_2HPO_4/0.5 M citric acid buffer, pH 5 and incubated for 1 minute at +37 °C. The concentration of iron was from 25 to 400 ng/ml. TMB (1 mg/ml in DMSO) was then added to 0.048 mg/ml. The solution was incubated at +37 °C for 1 more minute. Then H_2O_2 was added in the final concentration of 0.1 M. In the control samples deionized water was added. Cuvettes were immediately placed in a cell holder of a spectrophotometer, whose temperature was kept at +37 °C. Absorbance at 652 nm (A_{652}) was recorded every 5 sec after H_2O_2 addition over 60 seconds. The resulting absorbance was received by subtraction of the background absorbance at 652 nm caused by nanozymes themselves (samples without H_2O_2 addition)

The duration of the initial rate period was chosen with the criterion R2 close to 1 after a linear regression analysis. Specific activity was calculated using protocols developed by [**1**]

Storage stability of prussian blue nanoparticles

Storage stability of prussian blue nanoparticles with various sizes, synthesized at 10x scale was evaluated by DLS right after preparation, and then after 1, 3, and 5 months of storage at 4°C. After three months of storage, aggregation was observed in some samples: **R**, **R2C**, and **R2O**. We solved this problem by additional ultrasonication and centrifugation at a low speed (**Table S5**).

Measurement of the specific surface area

The specific surface area was evaluated by the BET method (low temperature inert gas adsorption) using a Sorbi-M (Meta, Russia) device. As a rule, before measuring the

specific surface, the samples are dried at a temperature of 200C to remove sorbed water. However, this process is of little use for Prussian blue nanoparticles, since they can begin to degrade when heated. We found that heating up to 200 degrees leads to a decrease in specific surface by 15–20%, so all samples were studied without drying.

Evaluation of gelatin A adsorption on the prussian blue nanoparticles

Prussian blue nanoparticles in the final concentration of 1 mg/ml were mixed with gelatin A in the mass ratio of 1:1, 1:2, 1:4, and 1:8 in deionized water. Samples were vortexed, briefly sonicated (probe diameter - 3 mm; amplification - 60%; duration - 10 s), incubated at +37 °C for 60 min on a rotational mixer (10 RPM), and centrifuged at 20,000 *g* until complete sedimentation. The concentration of gelatin in supernatants was measured by BCA assay. The size of nanoparticles after incubation with gelatin A was measured by DLS.

Long-term storage stability of nanoparticle conjugates

Conjugates PB/Gel A/BSA, PB/Gel A/Protein G, and PB/Gel A/MAb were stored at +4 °C in deionized water without stabilizers or preservatives. The concentration of prussian blue nanoparticles was between 1.03 and 1.16 mg/mL (concentration of coating protein is not taken into account). The size of nanoparticles was measured by DLS after the synthesis, then in 5 and 7 months.

Short-term colloidal stability of gelatin-coated prussian blue nanoparticles in comparison with non-coated ones

R2C and PB/Gel A/Protein G nanoparticles were diluted to 50 µg/mL in water and 0.2 M Na_2HPO_4/0.1 M citric acid (McIlvaine) buffer, pH 3–7. After 60 min of incubation, the size of nanoparticles was measured by DLS. Three technical replicates were measured for each sample.

Zeta-potential of gelatin-coated prussian blue nanoparticles

PB/Gel A/Protein G was diluted to 10 µg/mL in 5 mM Na_2HPO_4/citric acid buffer, pH 3.1-7. Zeta potential was measured by M3-PALS technique. Three technical replicates were measured for each sample.

Assessment of hydrolysis intensity of gelatin-coated prussian blue nanoparticles

PB/Gel A/BSA nanoparticles were diluted to 25 µg/mL in water, in 0.2 M Na_2HPO_2/0.1 M citric acid buffer (pH 2.5–8), in 0.1 M Glycine-HCl (pH 2 and 3), 0.1 M acetic acid-NaOH (pH 4 and 5), 0.1 M MES-NaOH (pH 6), and 0.1 M TRIS-HCl (pH 7 and 8). Nanoparticles were kept in plastic cuvettes in the dark humid chamber for 24 h at room temperature. One cuvette was prepared for each condition. Absorbance at 700 nm was measured after the dilution in the following time points: 15 min, 60 min, 120 min, and 24 h. Photos of cuvettes were taken before measurements. After 24 h, the size of nanoparticles was measured by DLS. Nanoparticles were resuspended by pipetting before measurement of absorbance and size if the sediment was observed

Additional results and discussion

Influence of various factors on the size of 'artificial peroxidase' nanoparticles

Syntheses were performed in 25 ml volume. The order of reagent addition was the same in all experiments: first, we added iron salts, then additives (citric or oxalic acids, potassium chloride, hydrochloric acid, potassium hydroxide), finally, the formation of prussian blue was initiated by the addition of hydrogen peroxide. Samples from the reaction medium were taken before and after the addition of H_2O_2, and then at 10, 30, 60, 90, 120, and 150 min. The following parameters were measured: size and polydispersity, zeta potential, and absorbance at 700 nm (A_{700}) (*in situ* samples). Then samples were

centrifuged, washed, and the above-mentioned parameters were measured again (centrifuged samples) to evaluate what nanoparticles would be obtained if the synthesis had been completed at the moment of sampling. The scheme of the experiment is given in Figure S2. Below some technological features of the 'artificial peroxidase' preparation process are listed.

Analysis of centrifuged samples obtained at 0 and 150 min time points revealed that the size of prussian blue nanoparticles is determined in the first minutes after the addition of hydrogen peroxide (Figures S18–S23). However, in the case of 3–4.5 mM of citric acid addition (Figure S22) enlargement of nanoparticles was observed for 30–60 min, probably due to assembling of smaller nanoparticles into large ones (see Section **"***Preparation of prussian blue nanoparticles with various sizes at 10× scale***"**).

In the course of synthesis, prussian blue nanoparticles usually form large loose aggregates. It allows centrifugation of formed prussian blue at relatively low speed, facilitating the purification process. Final ultrasound treatment leads to the destruction of these loose aggregates and obtaining nanoparticle dispersion. These nanoparticles can require a much higher centrifugation speed (30,000–40,000 *g* or more) for complete sedimentation. This feature may hinder the following functionalization process and should be taken into account. Conversely, in the course of synthesis at elevated temperature, the size of nanoparticles and their polydispersity gradually decreased.

An increase of nanoparticle concentration (A_{700}) occurred even after 150 min from the start of synthesis (Figure S24), therefore agitation can be potentially prolonged for several hours to increase the yield of nanoparticles.

Hydrolysis of nanozymes upon storage in various buffers

In the neutral and alkaline conditions hydrolysis of prussian blue occurs [**2**]. The mechanism of hydrolysis is the attack of Fe^{II}-CN-Fe^{III} bonds by hydroxyl ions [**3**]. This limitation forces researchers to develop various methods of improving the storage stability of prussian blue nanoparticles, such as storage in highly acidic conditions [**4**] or protection by nickel hexacyanoferrate [**5,6**]. Despite that, some authors report successful functionalization [**7**] or storage [**8,9**] of prussian blue nanoparticles in neutral and even alkaline mediums. Keeping in mind these discrepancies, we assessed the long-term stability of PB/Gel A/MAb at acidic and alkaline pH. .

Two types of buffers were used: 1) McIlvaine buffer which allows covering all desired range of pH values; 2) Range of buffers (glycine, acetate, MES, TRIS), which do not contain citric acid or phosphate ions to preclude the specific influence of these buffer components. Moreover, decoloration was almost complete in McIlvaine buffer, in TRIS-HCl buffer absorbance halved (Figures S11).

Undoubtedly, polymer coating and surface structure significantly affect the colloidal and structural stability of prussian blue nanoparticles [**10**]. Probably, some polymers can effectively protect prussian blue nanoparticles in alkaline medium, e.g. polyvinylpyrrolidone-coated nanoparticles were reported to withstand alkaline and neutral pH [**8**]. Nevertheless, the issue of stability of prussian blue nanoparticles at physiological pH (circa 7.4) and optimization of their long-term storage conditions are essential for their practical application and require more detailed study.

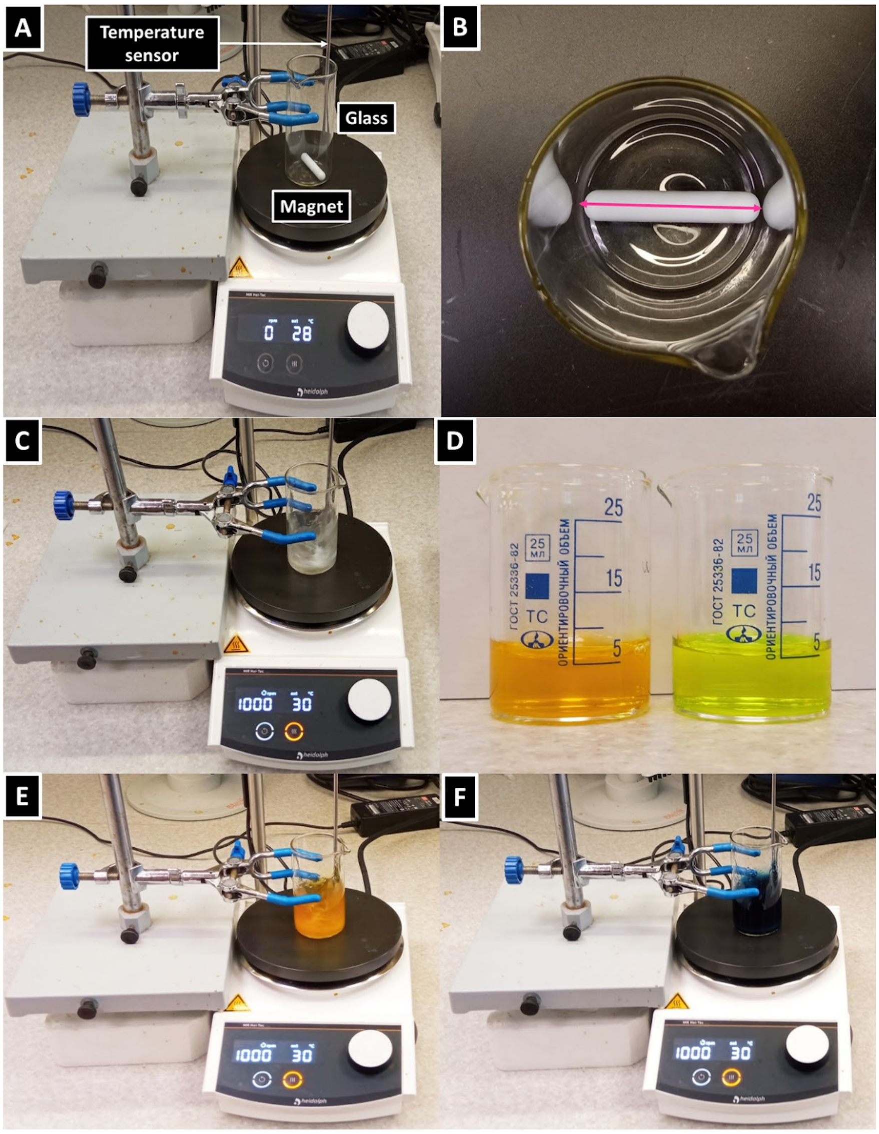

Figure S1. Synthesis of 'artificial peroxidase' prussian blue nanoparticles. (A) Experimental setup. (B) The stir bar matches the diameter of the beaker. (C) Pre-warming of the water before the addition of iron salts. (D) Concentrated solutions of $FeCl_3$ (left) and $K_3[Fe(CN)_6]$ (right). Mixture (25 ml) of $FeCl_3$ of $K_3[Fe(CN)_6]$ before (E) and after the addition of H_2O_2 (F).

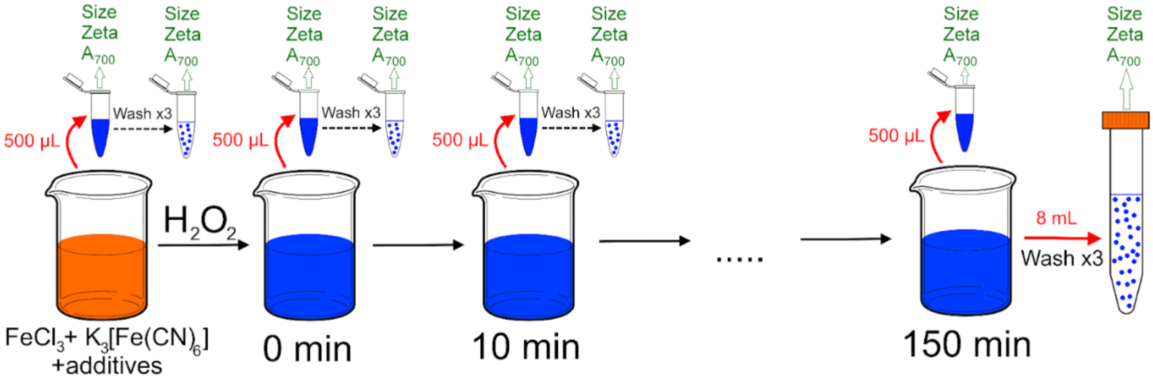

Figure S2. Influence of different factors on the synthesis of 'artificial peroxidase' nanozymes: design of the experiment.

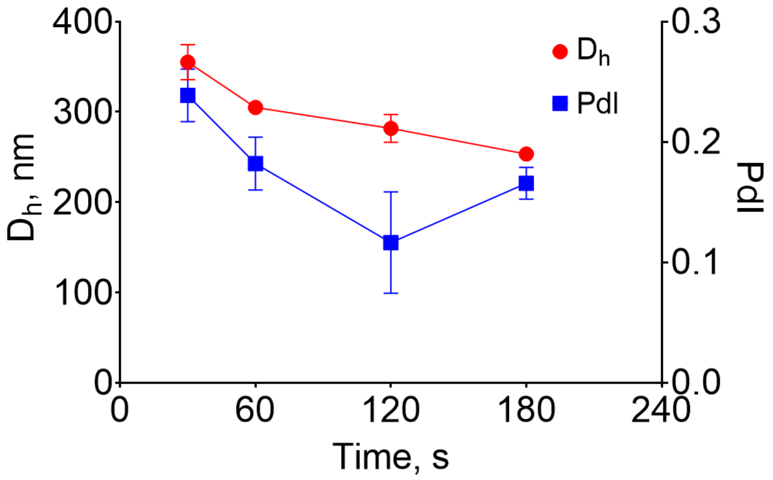

Figure S3. Effect of sonication time on the size and polydispersity of nanoparticles. Dh - hydrodynamic diameter, PdI - polydispersity index. n = 3, mean ± SD.

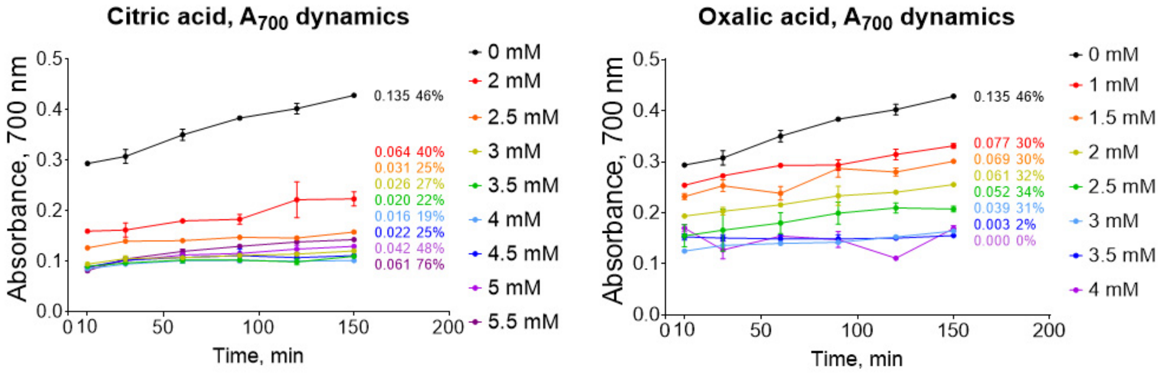

Figure S4. Change of A_{700} in the course of "artificial peroxidase" synthesis in the presence of various concentrations of citric and oxalic acids. The numbers to the right of the graphs show the difference in A_{700} between the time points of 10 and 150 minutes in absolute values and percentages. n = 3, mean ± SD.

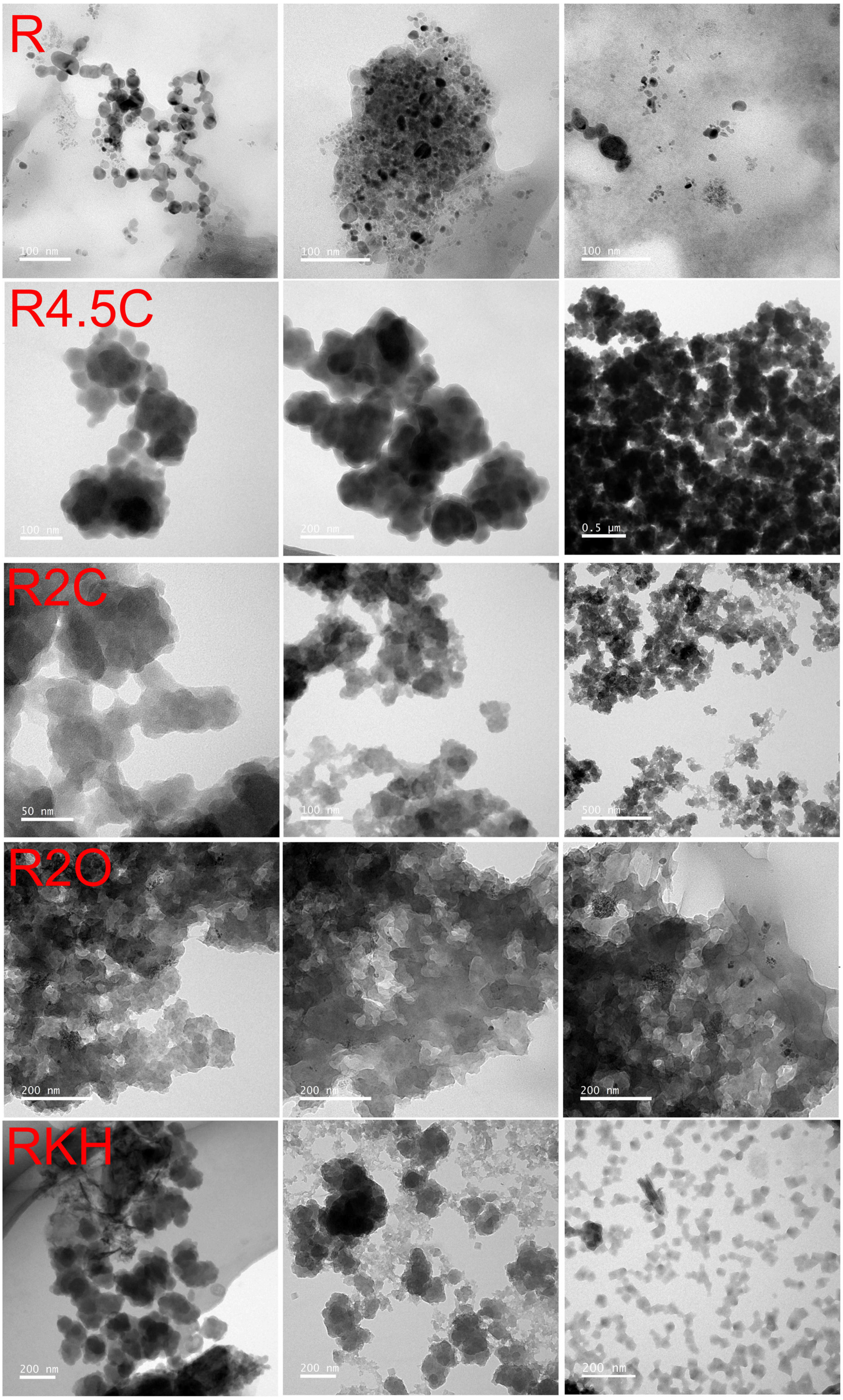

Figure S5. TEM images of prussian blue nanoparticles synthesized by the reductive approach ('artificial peroxidase'). Three images for each type of nanoparticles.

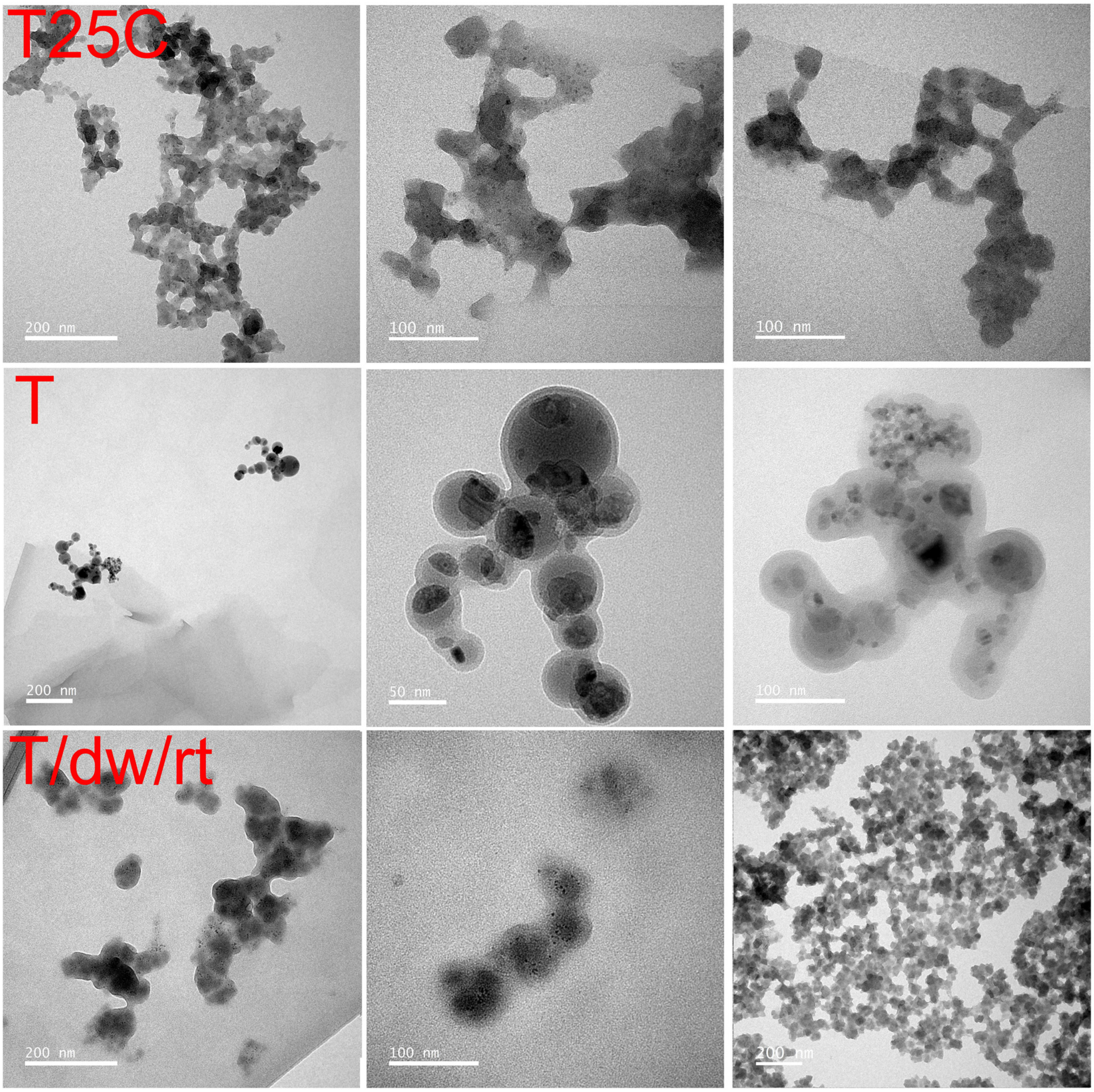

Figure S6. TEM images of prussian blue nanoparticles synthesized by the traditional approach. Three images for each type of nanoparticles.

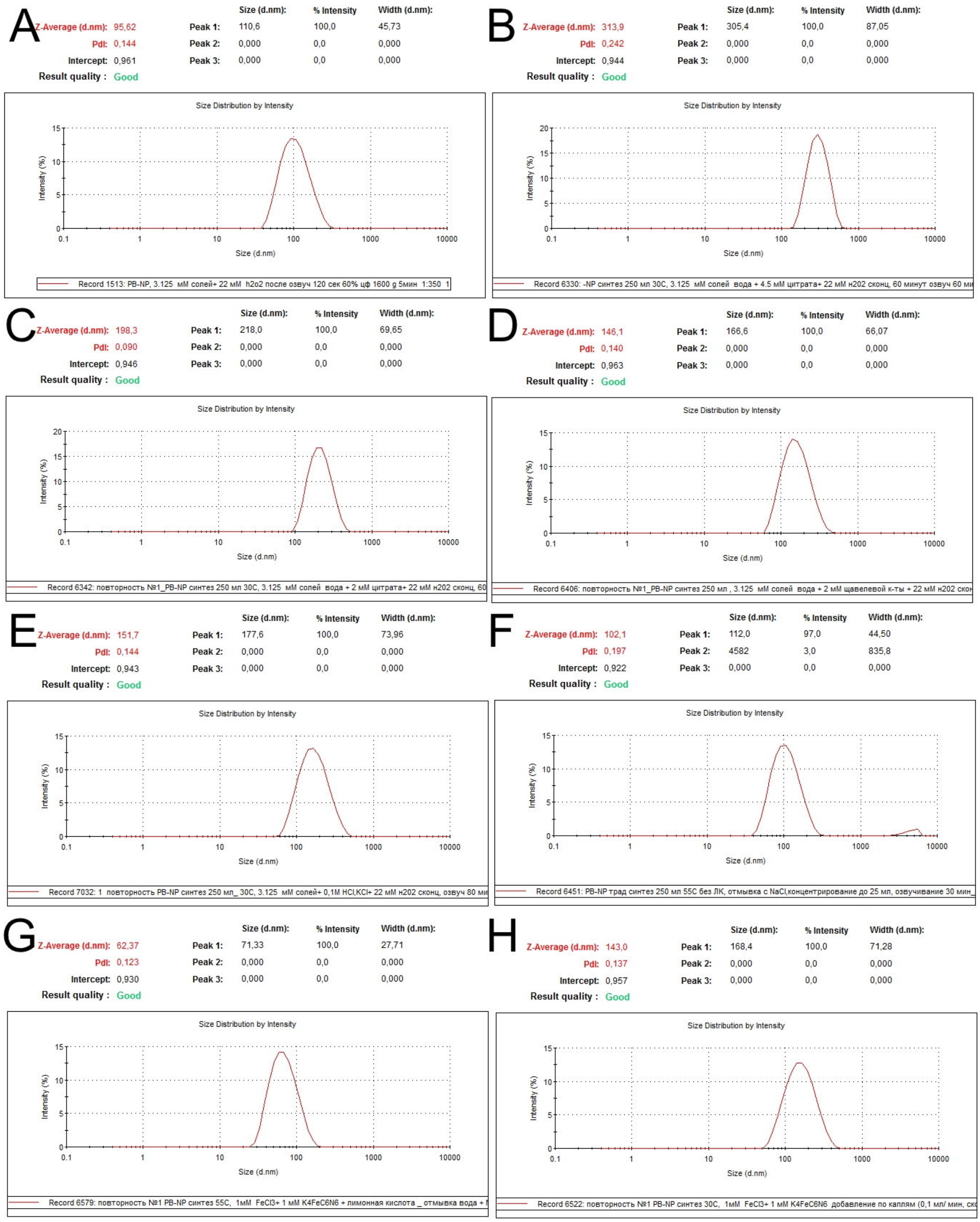

Figure S7. Typical intensity-weighted DLS size distribution plots for **R** (A), **R4.5C** (B), **R2C** (C), **R2O** (D), **RKH** (E), **T25C** (F), **T** (G), **T/dw/rt** (H).

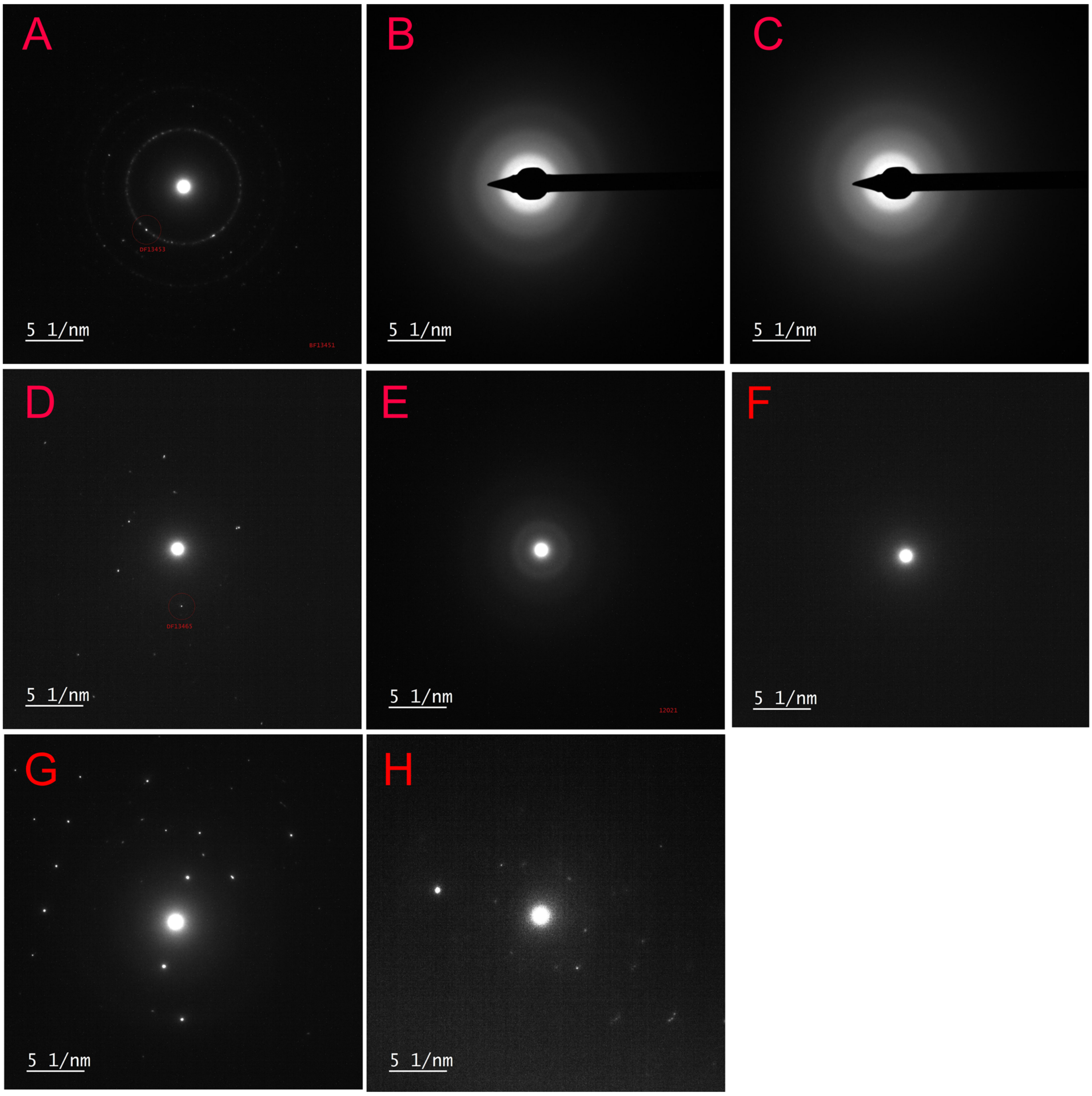

Figure S8. Selected area electron diffraction results for **R** (A), **R4.5C** (B), **R2C** (C), **R2O** (D), **RKH** (E), **T25C** (F), **T** (G), **T/dw/rt** (H).

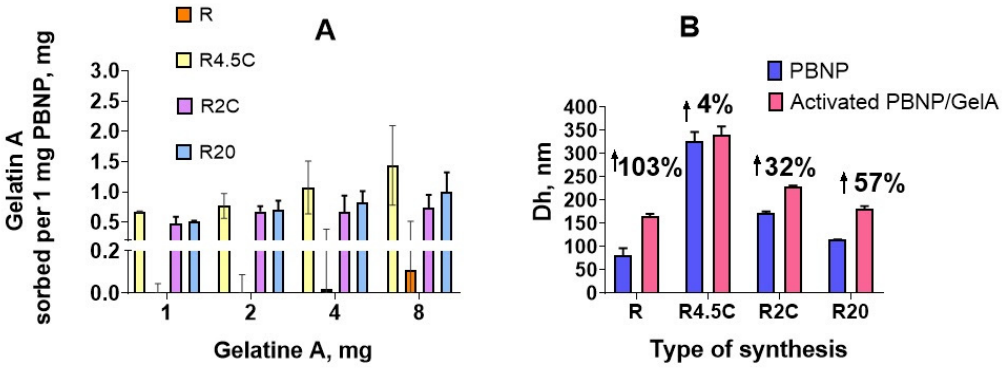

Figure S9. (A) Adsorption of gelatin A (180 bloom) on prussian blue nanoparticles prepared by reductive approach. (B) Change of hydrodynamic diameter (Dh) of nanoparticles after adsorption of gelatin and activation with glutaraldehyde. n = 3, mean ± SD.

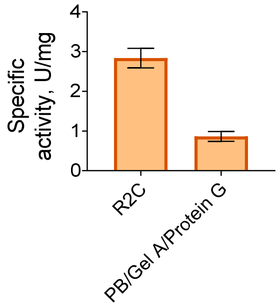

Figure S10. Specific activity of nanozymes before and after the functionalization, n = 3, mean ± SD. Mean values are given above the bars.

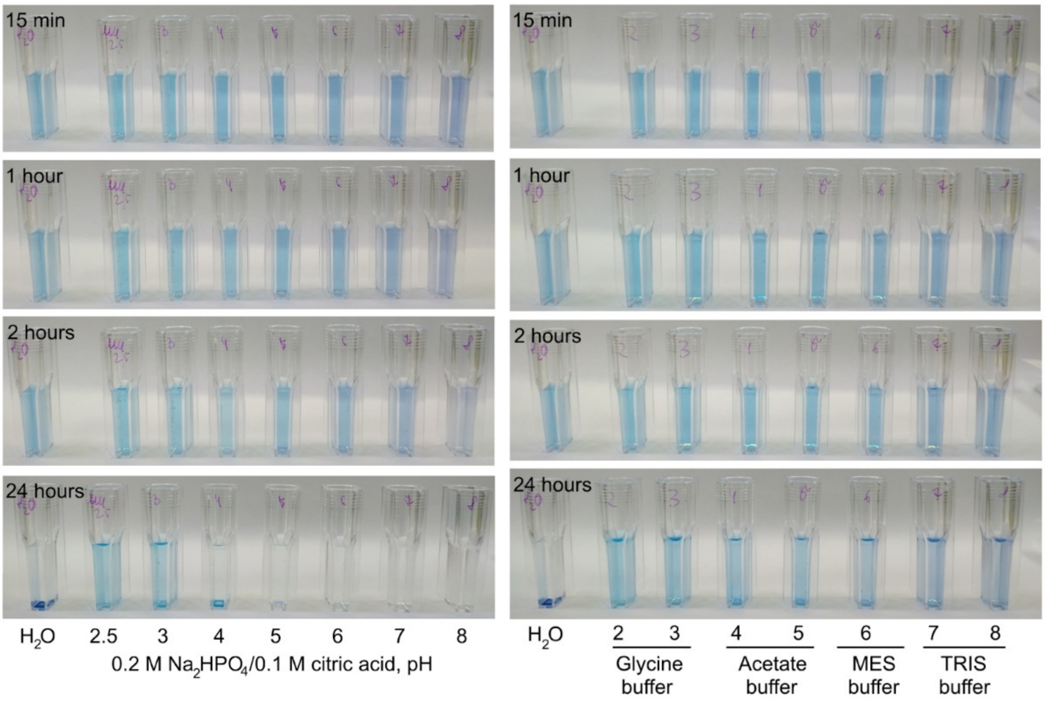

Figure S11. Color change of PB/Gel A/BSA diluted to 25 µg/mL in water and buffers with various pH (24 h of incubation). Left - McIlvaine buffer, right - 0.1 M Glycine-HCl, pH 2 and 3; 0.1 M acetic acid-NaOH, pH 4 and 5; 0.1 M MES-NaOH, pH 6; 0.1 M TRIS-HCl, pH 7 and 8. Size of nanoparticles is given in Figure S16. Change of absorbance at 700 nm is given in Figure S15.

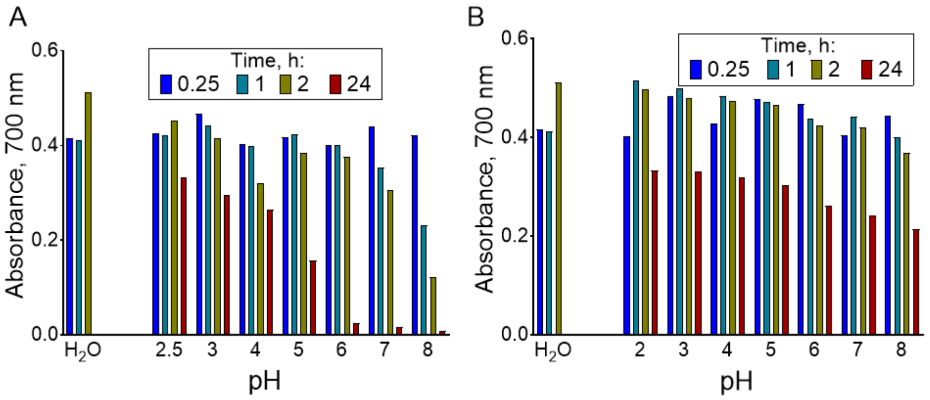

Figure S12. Absorbance at 700 nm of PB/Gel A/BSA diluted to 25 µg/mL in water and buffers with various pH (24 h of incubation). (A) - McIlvaine buffer, (B) - 0.1 M Glycine-HCl, pH 2 and 3; 0.1 M acetic acid-NaOH, pH 4 and 5; 0.1 M MES-NaOH, pH 6; 0.1 M TRIS-HCl, pH 7 and 8. N = 1.

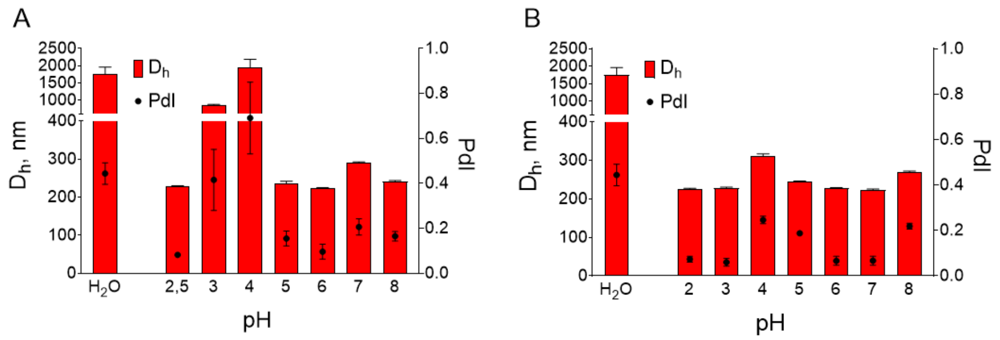

Figure S13. Size of PB/Gel A/BSA diluted to 25 μg/mL in water and buffers with various pH (24 h of incubation). (A) - McIlvaine buffer, (B) - 0.1 M Glycine-HCl, pH 2 and 3; 0.1 M acetic acid-NaOH, pH 4 and 5; 0.1 M MES-NaOH, pH 6; 0.1 M TRIS-HCl, pH 7 and 8. Dh - hydrodynamic diameter, PdI - polydispersity index. n = 3, mean ± SD.

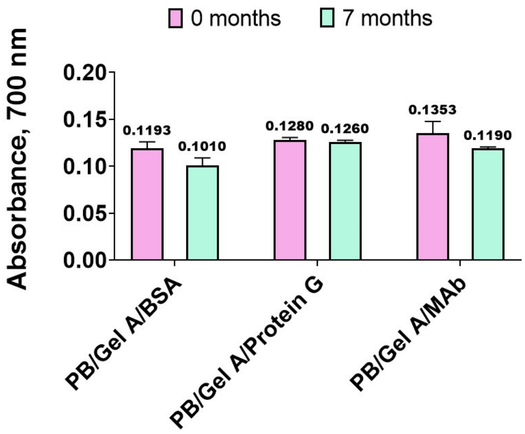

Figure S14. Change of absorbance at 700 nm of gelatin-coated prussian blue nanoparticles conjugated with affine molecules while storage at +4 °C, n = 3, mean ± SD. Mean values are given above the bars.

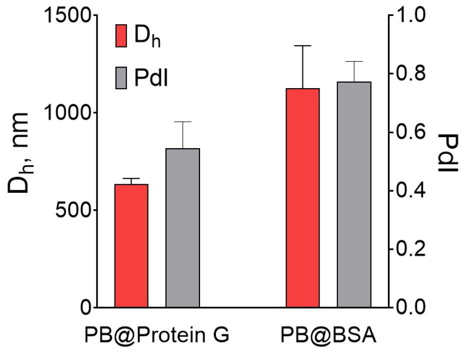

Figure S15. Size of prussian blue nanoparticles conjugated with protein G (PB@Protein G) or BSA (PB@BSA) via adsorption. Dh - hydrodynamic diameter, PdI - polydispersity index. n = 3, mean ± SD.

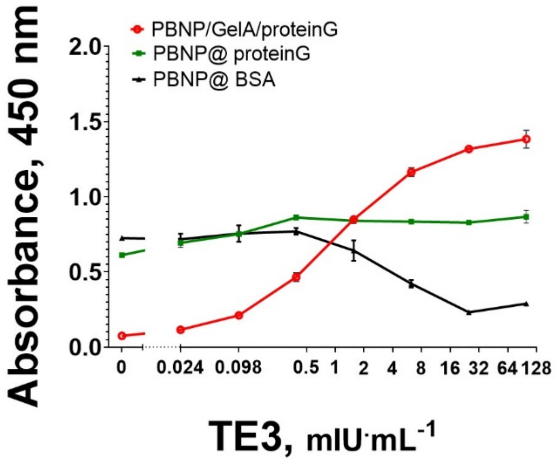

Figure S16. Calibration curves of anti-tetanus IgG (TE-3), which were obtained using nanozymes, synthesized by different methods: PBNP/GelA/protein G – protein G attached to gelatin-coated nanoparticles via glutaraldehyde; PBNP@protein G – protein G was directly adsorbed on prussian blue nanoparticles; PBNP@BSA – BSA was directly adsorbed on prussian blue nanoparticles (negative control). n = 3, mean ± SD. Substrate buffer: 0.1 M citrate-phosphate buffer, pH 5.

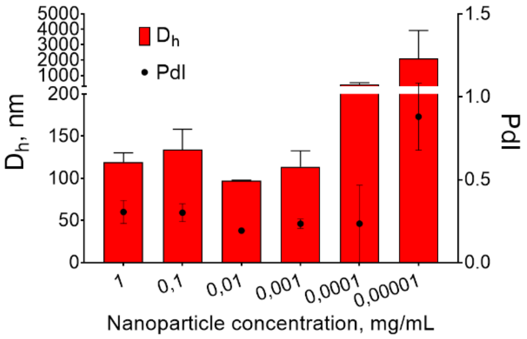

Figure S17. Relationship between nanoparticle concentration and DLS results. Dh - hydrodynamic diameter, PdI - polydispersity index. n = 3, mean ± SD.

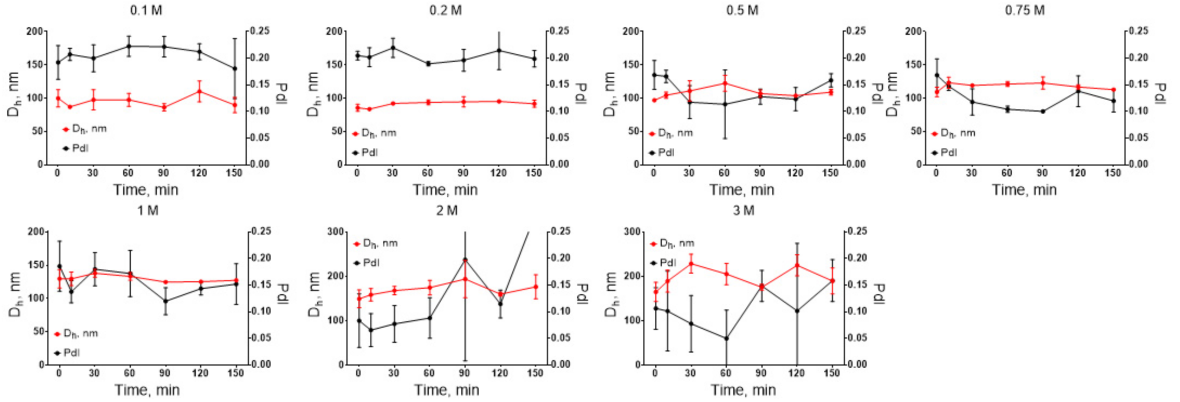

Figure S18. Change of size and polydispersity of nanoparticles in the course of synthesis. Centrifuged samples. Ionic strength experiment. Concentration of added KCl is above the graphs. Dh - hydrodynamic diameter, PdI - polydispersity index. n = 3, mean ± SD.

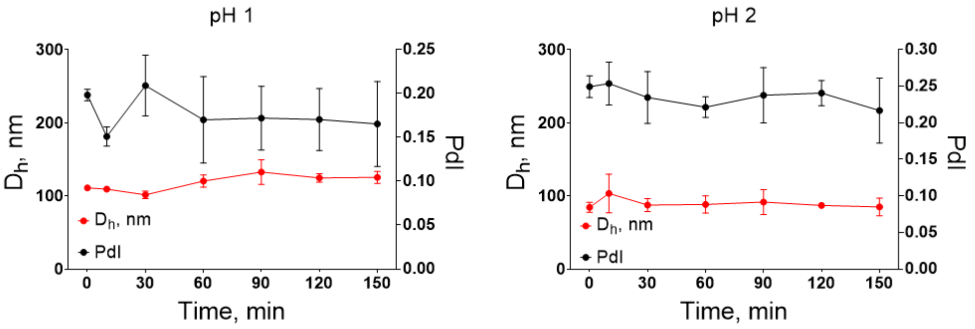

Figure S19. Change of size and polydispersity of nanoparticles in the course of synthesis. Centrifuged samples. pH experiment. pH value is above the graphs. Dh - hydrodynamic diameter, PdI - polydispersity index. n = 3, mean ± SD.

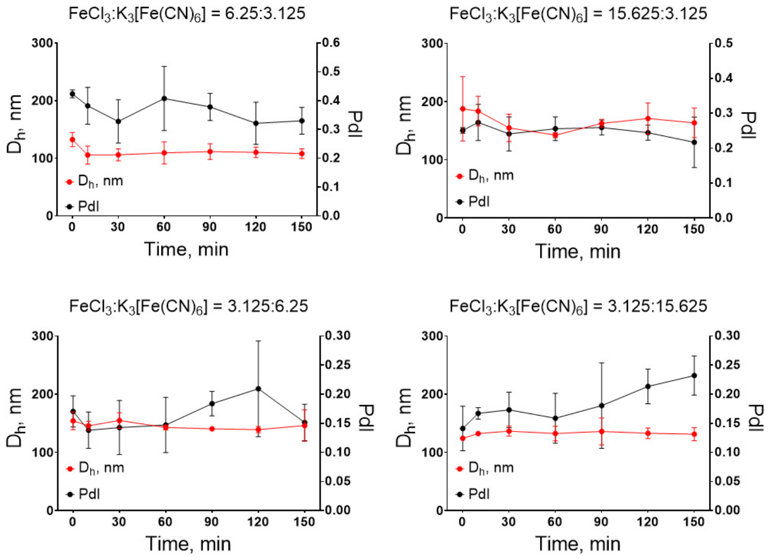

Figure S20. Change of size and polydispersity of nanoparticles in the course of synthesis. Centrifuged samples. Salt ratio experiment. $FeCl_3$:$K_3[Fe(CN)_6]$ ratio is above the graphs. Dh - hydrodynamic diameter, PdI - polydispersity index. n = 3, mean ± SD.

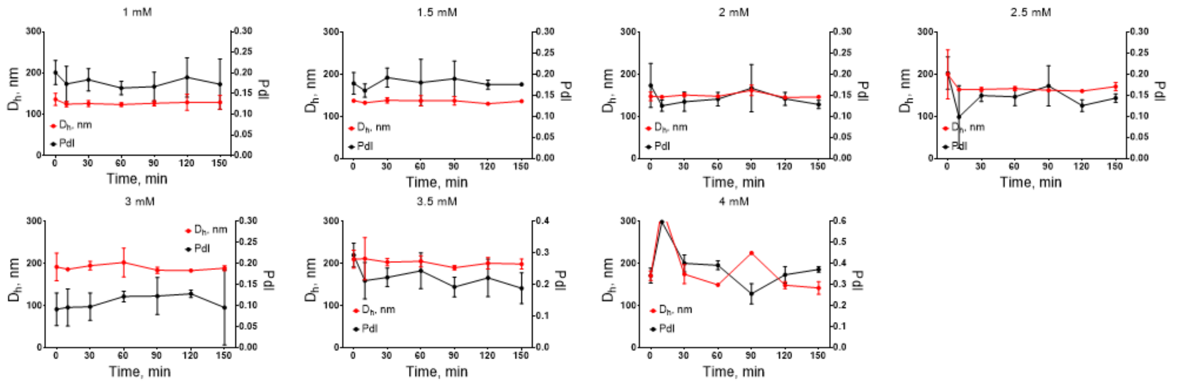

Figure S21. Change of size and polydispersity of nanoparticles in the course of synthesis. Centrifuged samples. Oxalic acid concentration experiment. Concentration of added oxalic acid is above the graphs. Dh - hydrodynamic diameter, PdI - polydispersity index. n = 3, mean ± SD.

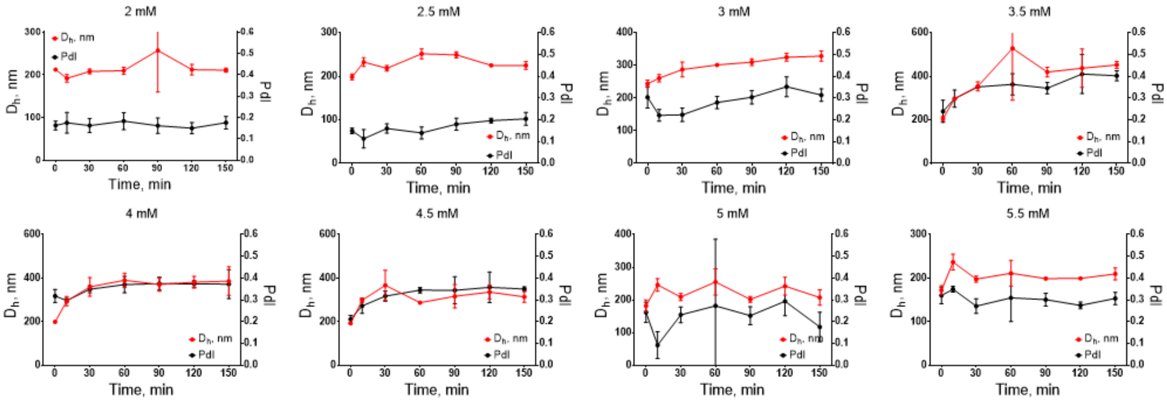

Figure S22. Change of size and polydispersity of nanoparticles in the course of synthesis. Centrifuged samples. Citric acid concentration experiment. Concentration of added citric acid is above the graphs. Dh - hydrodynamic diameter, PdI - polydispersity index. n = 3, mean ± SD.

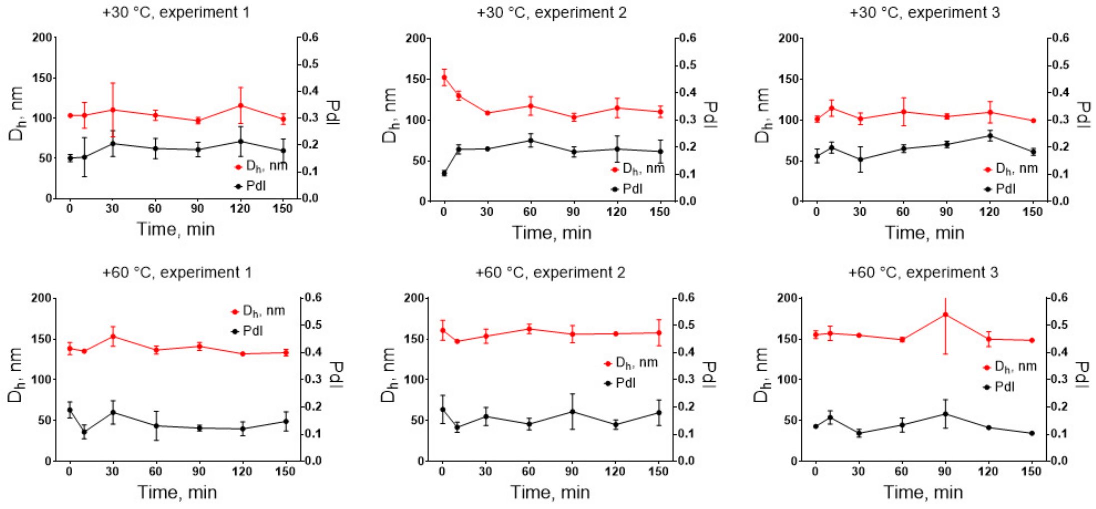

Figure S23. Change of size and polydispersity of nanoparticles in the course of synthesis. Centrifuged samples. Temperature experiment (three identical experiments were performed for each temperature). Temperature and number of experiment are above the graphs. Dh - hydrodynamic diameter, PdI - polydispersity index. n = 3, mean ± SD.

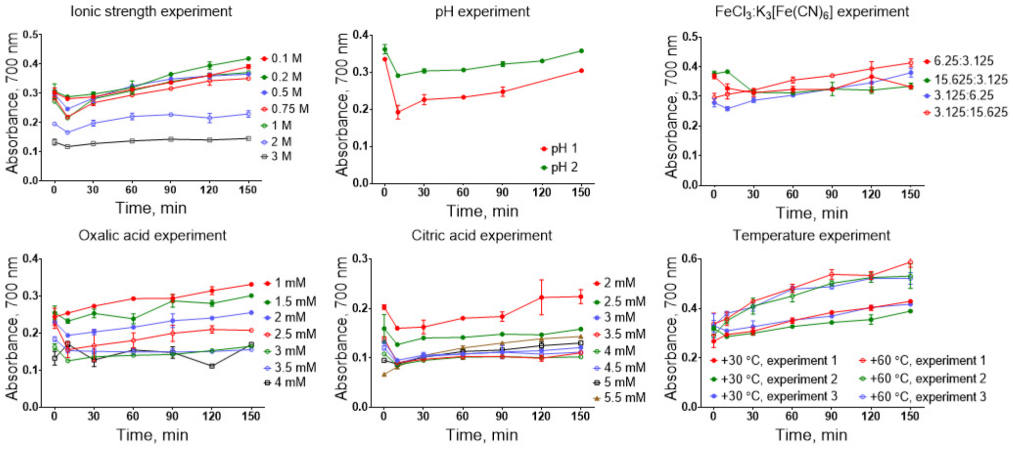

Figure S24. Change of absorbance at 700 nm in the course of synthesis. *In situ* samples. Type of experiment is above the graphs. n = 3, mean ± SD.

Table S1. Hydrodynamic diameters (Dh) and polydispersity (PdI) of prussian blue nanoparticles synthesized in different conditions at 10× scale.

Type		№1			№2			№3			
	Target[a]	D_h, nm	PdI	Diff.	D_h, nm	PdI	Diff.	D_h, nm	PdI	Diff.	CV, %
R	101.3; 0.119	96,3± 8,8	0,19± 0,05	-5%	92,9± 0,005	0,159± 0,001	-9%	97,7± 0,6	0,125± 0,007	-4%	2,6
R4.5C	318.6; 0.22	355,6± 4,9	0,15± 0,04	+11%	330,± 1,9	0,264± 0,01	+4%	323,5± 2,2	0,19± 0,02	+1%	1,4
R2C	185.5; 0.08	202± 0,2	0,14± 0,07	+9%	202,9,± 0.9	0,19± 0,06	+9%	203,4± 2,6	0,102± 0,02	+10%	0,3
R2O	145.6; 0.12	135± 0,75	0,17± 0,08	-7%	133,± 0,7	0,14± 0,02	-9%	134,4± 1,35	0,169± 0,07	-8%	0,8
RKH	-	194,1± 1,98	0,13± 0,01	-	203,± 0,7	0,109± 0,02	-	202,6± 2,4	0,12± 0,02	-	2,5
T25C	54.04; 0.15	67,18± 0,38	0,16± 0,02	+24%	75,0± 1,73	0,15± 0,015	+38%	68,3± 1,6	0,14± 0,024	+26%	6,1
T	84.6; 0.2	89,85± 0,7	0,13± 0,001	+6%	91,1± 1,4	0,14± 0,031	+8%	91,21± 0,3	0,116± 0,02	+8%	0,9
T/dw/rt	135.8; 0.14	126,7± 0,34	0,12± 0,004	-7%	123± 0,7	0,11± 0,01	-9%	-	-	-	2,1

[a]Target – target Dh and PdI of nanoparticles predicted from experiments in a lower reaction volume. Diff. – size difference between obtained and target size. CV – coefficient of variation of Dh of three batches.

Table S2. Specific activity (U/mgFe) of prussian blue nanoparticles synthesized by different methods. Mean of 3 technical replicates is reported for each batch.

	R	R4.5C	R2C	R2O	RKH	T25C	T	T/dw/rt
Batch 1	2.463	1.470	2.813	3.203	2.171	2.616	2.273	1.896
Batch 2	2.376	1.660	2.566	3.130	2.052	2.316	2.013	1.810
Batch 3	2.543	1.583	2.836	3.066	2.093	2.79	1.900	-
Mean	2.461	1.571	2.739	3.133	2.105	2.574	2.062	1.853
Standard deviation	0.083	0.095	0.149	0.068	0.060	0.239	0.191	0.061

Table S3. Comparison of specific activity (U/mgFe) of prussian blue nanoparticles synthesized by different methods. One-way ANOVA with Sidak's post-hoc test. n = 3 (for T/dw/rt n = 2).

Compared samples	Mean specific activity of group 1	Mean specific activity of group 2	p value
R vs. R4.5C	2.461	1.571	< 0.0001
R vs. R2C	2.461	2.739	0.5244
R vs. R2O	2.461	3.133	0.0007
R vs. RKH	2.461	2.105	0.1669
R vs. T25C	2.461	2.574	> 0.9999

R vs. T	2.461	2.062	0.0797
R vs. T/dw/rt	2.461	1.853	0.0062
R4.5C vs. R2C	1.571	2.739	< 0.0001
R4.5C vs. R2O	1.571	3.133	< 0.0001
R4.5C vs. RKH	1.571	2.105	0.0073
R4.5C vs. T25C	1.571	2.574	< 0.0001
R4.5C vs. T	1.571	2.062	0.0156
R4.5C vs. T/dw/rt	1.571	1.853	0.6877
R2C vs. R2O	2.739	3.133	0.0861
R2C vs. RKH	2.739	2.105	0.0014
R2C vs. T25C	2.739	2.574	0.9936
R2C vs. T	2.739	2.062	0.0007
R2C vs. T/dw/rt	2.739	1.853	0.0001
R2O vs. RKH	3.133	2.105	< 0.0001
R2O vs. T25C	3.133	2.574	0.0048
R2O vs. T	3.133	2.062	< 0.0001
R2O vs. T/dw/rt	3.133	1.853	< 0.0001
RKH vs. T25C	2.105	2.574	0.023
RKH vs. T	2.105	2.062	> 0.9999
RKH vs. T/dw/rt	2.105	1.853	0.8417
T25C vs. T	2.574	2.062	0.0107
T25C vs. T/dw/rt	2.574	1.853	0.0011
T vs. T/dw/rt	2.062	1.853	0.9702

Table S4. Hydrodynamic diameters of prussian blue nanoparticles after 1, 3, and 5 months of storage at +4 °C.

	**R4.5C**		**R2C**		**R2O**		**R**		**RKH**		**T**		**T/dw/rt**		**T25C**	
	**Dh, nm**	**PDI**	**Dh, nm**	**PDI**	**Dh, nm**	**PDI**	**Dh, nm**	**PDI**	**Dh, nm**	**PDI**	**Dh, nm**	**PDI**	**Dh, nm**	**PDI**	**Dh, nm**	**PDI**
	348.9	0.187	202	0.073	**179.8**	**0.281**	90.68	0.15	196	0.137	88.84	0.143	126.6	0.133	66.67	0.128
**1**	360.6	0.095	202.4	0.118	135.2	0.11	89.52	0.166	195	0.132	90.32	0.121	127.2	0.123	67.29	0.175
	357.2	0.172	**314.7**	**0.235**	136.7	0.116	108.8	0.26	191.4	0.114	90.39	0.126	126.4	0.126	67.59	0.179
	330.5	0.268	202	0.206	134.3	0.162	92.91	0.158	203.9	0.125	90.42	0.189	124.5	0.107	77.46	0.168
**2**	328.7	0.279	**295.6**	**0.263**	133.6	0.118	**139.6**	**0.283**	202.3	0.122	89.84	0.134	123	0.125	73.67	0.131
	333.4	0.245	203.8	0.102	132.5	0.137	92.92	0.16	203.6	0.08	93.02	0.107	123.2	0.1	73.94	0.151
	322.4	0.217	202.9	0.078	**193.8**	**0.264**	98.52	0.13	200.4	0.107	91.41	0.135			67.42	0.126
**3**	321.6	0.182	200.5	0.098	136	0.113	97.26	0.13	201.4	0.154	91.45	0.087			66.95	0.121
	326.6	0.181	206.9	0.13	133.3	0.13	97.28	0.116	205.9	0.113	90.77	0.126			70.54	0.174

**1 month**																
	366.9	0.214	205.5	0.092	132.2	0.136	90.18	0.151	200.4	0.156	88.38	0.128	127	0.131	61.97	0.149
**1**	367.8	0.312	204.7	0.135	132.5	0.132	88.96	0.149	206.5	0.186	88.18	0.138	125.1	0.134	62.61	0.093
	370.6	0.233	**313.3**	**0.232**	134.4	0.123	**121.4**	**0.276**	203.3	0.164	135.1	0.257	125.5	0.09	62.37	0.145
	325.8	0.243	204.7	0.087	130.5	0.131	92.84	0.157	208.3	0.121	91.26	0.15	**202.2**	**0.258**	71.53	0.143
**2**	330.4	0.243	205.1	0.121	128.2	0.102	92.06	0.145	**290.2**	**0.232**	90.17	0.11	123.5	0.124	71.33	0.136
	324.4	0.28	203.8	0.107	128.8	0.13	90.97	0.152	211.3	0.099	91.61	0.138	122.1	0.13	72.7	0.163
	321.7	0.264	212	0.109	130.3	0.16	99.62	0.125	**307.5**	**0.262**	92.98	0.149			66.32	0.143
**3**	514.6	0.416	208.3	0.079	129.2	0.116	**160.9**	**0.254**	205	0.156	91.6	0.147			103.5	0.249
	316.8	0.258	212	0.09	211.3	0.264	99.36	0.129	207.1	0.123	**152.5**	**0.245**			64.81	0.125
**3 months**																

	341.2	0.199	169.1	0.094	117.3	0.159	65.51	0.125	197	0.123	90.31	0.152	126.3	0.1	64.72	0.127
**1**	347.1	0.229	172.8	0.086	**162.5**	**0.259**	66.75	0.142	192.2	0.168	**142**	**0.242**	125.9	0.121	65.37	0.14
	344.6	0.161	170.7	0.096	113.9	0.101	68.05	0.143	**235**	**0.241**	86.89	0.117	126.5	0.153	68.21	0.189
	331	0.267	172.1	0.107	116.6	0.113	101.9	0.245	198.6	0.133	92.87	0.166	122.3	0.126	72.24	0.159
**2**	481.6	0.38	177.9	0.07		0.227	**114**	0.251	**235**	**0.268**	95.33	0.15	121.7	0.123	72.22	0.123
	325.9	0.231	178.1	0.118	113.4	0.112	83.76	0.098	199.9	0.129	90.42	0.132	119.2	0.124	71.72	0.132
	320.8	0.246	172.4	0.093	159.5	0.256	90.23	0.102	196.9	0.092	92.18	0.133			66.87	0.166
**3**	327.2	0.263	172.9	0.095	135.6	0.231	88.33	0.114	194.4	0.115	90.23	0.117			65.87	0.109
	319.1	0.251	169.9	0.099	115.3	0.093	**122.4**	**0.245**	197.3	0.136	93.48	0.145			64.57	0.104
**5 months**																		
	346.1	0.207	171.1	0.104	118.9	0.167	65.33	0.152	185.7	0.122	87.16	0.125	132.9	0.134	63.98	0.14

**1**	351	0.239	170.8	0.083	113.2	0.089	64.51	0.138	182.1	0.124	89.35	0.156	129.7	0.162	63.42	0.133
	346.5	0.208	169.8	0.074	114.9	0.105	63.39	0.129	184.1	0.129	**121.9**	**0.249**	131.3	0.123	66.33	0.186
	305.6	0.231	174.7	0.117	113.4	0.095	82.05	0.117	192.2	0.097	90.88	0.132	121.2	0.141	71	0.142
**2**	309.3	0.281	173.6	0.09	115	0.13	81.22	0.129	194	0.067	93.69	0.128	122	0.123	72.95	0.133
	309.5	0.221	178.5	0.072	116.5	0.131	**120.4**	**0.254**	193.3	0.092	89.6	0.145	**151.6**	**0.244**	71.63	0.151
	308.1	0.212	172.9	0.064	114	0.116	**119**	**0.174**	194.5	0.123	90.56	0.114			62.69	0.117
**3**	307.3	0.217	169.3	0.1	113.2	0.097	88.24	0.116		0.212	88.89	0.138			63.45	0.122
	312.3	0.251	169.5	0.089	113.9	0.095	86.55	0.12	195.1	0.092	88.92	0.116			57.32	0.38

Dh - hydrodynamic diameter, PdI - polydispersity index. Bold type indicate values, which were excluded from calculation of nanoparticle mean size and polydispersity index. We observed between-replicate inconsistencies when the size of nanoparticles was measured by DLS. Usually, one of three technical replicates had a size significantly different from two others (larger size and polydispersity), which is explained by the high sensitivity of the DLS method to the presence of aggregates [11]. These replicates were not taken into account when the analysis was performed.

Table S5. Details of additional manipulation with aggregated batches of prussian blue nanoparticles after three months of storage at +4 °C.

Type of nanoparticles	Time of additional ultrasonication (60% amplification), min	Time of additional centrifugation (500 g), min	Time of additional centrifugation (1600 g), min	Time of additional centrifugation (4500 g), min
**R**	20	-	10	45
**R2C**	-	-	75	45
**R2O**	20	35	60	60

References

1. Jiang, B.; Duan, D.; Gao, L.; Zhou, M.; Fan, K.; Tang, Y.; Xi, J.; Bi, Y.; Tong, Z.; Gao, G. F.; Xie, N.; Tang, A.; Nie, G.; Liang, M.; Yan, X. Standardized assays for determining the catalytic activity and kinetics of peroxidase-like nanozymes. *Nat. Protoc*. **2018**, *13*, 1506–1520. https://doi.org/10.1038/s41596-018-0001-1.
2. Karyakin, A. A. Advances of Prussian blue and its analogues in (bio)sensors. *Curr. Opin. Electrochem*. **2017**, *5*, 92–98. https://doi.org/10.1016/j.coelec.2017.07.006.
3. Cano-Mejia, J.; Burga, R. A.; Sweeney, E. E.; Fisher, J. P.; Bollard, C. M.; Sandler, A. D.; Cruz, C.; Fernandes, R. Prussian blue nanoparticle-based photothermal therapy combined with checkpoint inhibition for photothermal immunotherapy of neuroblastoma. *Nanomed.: Nanotechnol. Biol. Med.* **2017**, *13*, 771–781. https://doi.org/10.1016/j.nano.2016.10.015.
4. Komkova, M. A.; Karyakina, E. E.; Karyakin, A. A. Catalytically synthesized Prussian Blue nanoparticles defeating natural enzyme peroxidase. *J. Am. Chem. Soc.* **2018**, *140*, 11302–11307. https://doi.org/10.1021/jacs.8b05223.
5. Ishizaki, M.; Ohshida, E.; Tanno, H.; Kawamoto, T.; Tanaka, H.; Hara, K.; Kominami, H.; Kurihara, M. H_2O_2-sensing abilities of mixed-metal (Fe-Ni) Prussian blue analogs in a wide pH range. *Inorg. Chim. Acta* **2020**, *502*, 119314. https://doi.org/10.1016/j.ica.2019.119314.
6. Karpova, E. V.; Shcherbacheva, E. V.; Komkova, M. A.; Eliseev, A. A.; Karyakin, A. A. Core–Shell Nanozymes "Artificial Peroxidase": Stability with Superior Catalytic Properties. *J. Phys. Chem. Lett.* **2021**, *12*, 5547–5551. https://doi.org/10.1021/acs.jpclett.1c01200.
7. Liu, Z.; Hua, Q.; Wang, J.; Liang, Z.; Zhou, Z.; Shen, X.; Lei, H.; Li, X. Prussian blue immunochromatography with portable smartphone-based detection device for zearalenone in cereals. *Food Chem*. **2022**, *369*, 131008. https://doi.org/10.1016/j.foodchem.2021.131008.
8. Xu, Y.; Zhang, Y.; Cai, X.; Gao, W.; Tang, X.; Chen, Y.; Chen, J.; Chen, L.; Tian, Q.; Yang, S.; Zheng, Y.; Hu, B. Large-scale synthesis of monodisperse Prussian blue nanoparticles for cancer theranostics via an "in situ modification" strategy. *Int. J. Nanomed.* **2018**, *14*, 271–288. https://doi.org/10.2147/IJN.S183858.
9. Ren, J.; Su, L.; Hu, H.; Yin, X.; Xu, J.; Liu, S.; Wang, J.; Wang, Z.; Zhang, D. Expanded detection range of lateral flow immunoassay endowed with a third-stage amplifier indirect probe. *Food Chem*. **2021**, *377*, 131920. https://doi.org/10.1016/j.foodchem.2021.131920.
10. Wang, Z.; Long, Y.; Fan, J.; Xiao, C.; Tong, C.; Guo, C.; Chen, X.; Liu, B.; Yang, X. Biosafety and biocompatibility assessment of Prussian blue nanoparticles in vitro and in vivo. *Nanomedicine (London, U.K.)* **2020**, *15*, 2655–2670. https://doi.org/10.2217/nnm-2020-0191.
11. Langevin, D.; Raspaud, E.; Mariot, S.; Knyazev, A.; Stocco, A.; Salonen, A.; Luch, A.; Haase, A.; Trouiller, B.; Relier, C.; Lozano, O.; Thomas, S.; Salvati, A.; Dawson, K. Towards reproducible measurement of nanoparticle size using dynamic light scattering: Important controls and considerations. *NanoImpact* **2018,** *10*, 161–167. https://doi.org/10.1016/j.impact.2018.04.002.
